# Risk factors of congenital anomalies of the kidney and urinary tract (CAKUT): Exposure to mobile phones during pregnancy

**DOI:** 10.55730/1300-0144.5790

**Published:** 2023-11-18

**Authors:** Kübra ÇELEĞEN, Esra ÖZGÜL, Zeynep YEŞİLDAĞ, Erdem Yusuf ÇAMIRCI, Mehmet ÇELEĞEN, Aysegül BÜKÜLMEZ

**Affiliations:** 1 Division of Pediatric Nephrology, Department of Pediatrics, Afyonkarahisar Health Sciences University Faculty of Medicine, Afyonkarahisar, Turkiye; 2 Department of Radiology, Afyonkarahisar Health Sciences University Faculty of Medicine, Afyonkarahisar, Turkiye; 3 Department of Pediatrics, Afyonkarahisar Health Sciences University Faculty of Medicine, Afyonkarahisar, Turkiye

**Keywords:** Congenital anomalies of the kidney and urinary tract, specific absorption rate, mobile phone, electromagnetic field, pregnancy

## Abstract

**Background/aim:**

Congenital anomalies of the kidney and urinary tract(CAKUT) are the leading causes of childhood chronic kidney disease (CKD). The etiology of most of the cases is thought to be multifactorial. In this study, risk factors for CAKUT and the effect of mobile phone-related electromagnetic field (EMF) exposure during pregnancy were investigated.

**Materials and methods:**

Fifty-seven cases and 57 healthy controls under 2 years of age were included and their mothers were subjected to a questionnaire. Groups were compared for parents’ demographics, pregestational (chronic disease, body mass index, use of the folic acid supplements) and antenatal variables (gestational disease, weight gain during pregnancy,) and exposures during pregnancy. To assess mobile phone-related radiation exposure, all participants were asked about their daily call time, the proximity of the phone when not in use, and the models of their mobile phones. The specific absorption rate (SAR) of the mobile phones and the effective SAR value (SAR × call time) as an indicator of EMF exposure were recorded.

**Results:**

Excess weight gain according to BMI during pregnancy was related to an increased risk of CAKUT (p=0.012). Folic acid use before pregnancy was protective for CAKUT (p = 0.028). The call time of mothers of the CAKUT group was significantly longer than the control (p = 0.001). An association was observed between higher effective SAR values and increased risk of CAKUT (p = 0.03). However the proximity of the mobile phone to the mother’s body when not in use was not found as a risk factor.

**Conclusion:**

The etiology of CAKUT is multifactorial. Our results suggest that prolonged phone call and higher EMF exposure during pregnancy increases the risk of CAKUT in the offspring.

## 1. Introduction

Congenital anomalies of the kidney and urinary tract (CAKUT) are a cluster of abnormalities with atypical development of the kidney, ureters, bladder, and urethra. CAKUT constitutes almost 20%–30% of all anomalies identified during the prenatal period [1]. The whole prevalence of CAKUT has been reported to be approximately 35:10,000 births in the European Union [2]. CAKUT plays a causative role in 40%–50% of pediatric and 7% of adult cases with end-stage renal disease worldwide [3]. However, the etiology of most cases of CAKUT is unclear. Monogenic mutations were reported to be detected in approximately 5%–20% of CAKUT cases. Therefore, most of the causes of CAKUT are thought to be multifactorial, including pregestational and gestational factors, in-utero environments, and genetics [4–5]. Although there are conflicting results, risk factors reported in the literature are gestational diabetes mellitus, maternal thalassemia, maternal obesity, smoking, fertility treatment, and low birth weight of the offspring [5–7].

Electronic devices have become an indispensable part of human life with the advancement of technology. However, electronic devices, such as mobile phones, televisions, and computers emit nonionizing electromagnetic field (EMF) radiation at a high-frequency level (100 kHz–300 GHz) [8, 9]. EMF radiation is reported to increase with the duration of use, the number of mobile devices used average call time, and the proximity of the EMF radiation to the body [8]. Maternal EMF exposure was reported to be related to miscarriage, the risks of giving birth prematurely, speech disabilities, hyperactivity of the offspring, and the development of congenital heart disease [9–13].

The effect of EMF on the development of the kidney and urinary tract is limited to several animal studies. Bedir et al. reported that EMF exposure produced by mobile phones within the first 20 days of pregnancy in rats resulted in renal congestion, dilatation of the Bowman capsule, and tubular defects with increased apoptosis [14]. However, there is no study in the literature investigating the relationship between EMF exposure during pregnancy and the development of CAKUT. We designed a case-control study to investigate the possible risk factors of CAKUT and the effects of EMF exposure to mobile phones during pregnancy.

## 2. Materials and methods

We conducted a case-control study to assess the pregestational and gestational risk factors for the occurrence of CAKUT. Patients under two years of age who were diagnosed with CAKUT in the Department of Pediatric Nephrology between January 2021 and May 2022 were included in the study. Patients were diagnosed with renal dysplasia, unilateral renal agenesis, multicystic dysplastic kidney, renal fusion anomalies, ectopic kidney, vesicoureteral reflux, posterior urethral valve, duplex collecting system, ureteropelvic junction (UPJ) obstruction, and ureterovesical junction (UVJ) obstruction. Renal diseases were confirmed by ultrasonography, voiding cystourethrography, and scintigraphy. Family consent was obtained for the study. The control group was selected from under 2-year-old individuals who needed abdominal imaging in their routine controls and did not have any kidney disease. The study was approved by the Ethics Committee of our institution.

Mothers of the patients were asked to fill out a questionnaire about demographics, socioeconomic, obstetric characteristics, medical and reproductive histories, and exposures during pregnancy. Maternal and paternal age (<20, 20–34, ≥35), maternal education level (low, intermediate, high), mode of delivery, gravidity history, and duration of last delivery were recorded as covariates. The potential risk factors evaluated for this study were preconception (chronic disease, body mass index, infertility treatment, use of folic acid supplements) and antenatal risk factors (gestational disease, weight gain during pregnancy use of vitamins, taking additional medicine, maternal smoking, and passive smoking) and maternal exposures to electronic devices during pregnancy.

BMI is categorized based on the World Health Organization [15]. During pregnancy, weight gain was categorized as excessive if it exceeded the Institute of Medicine recommendations based on prepregnancy BMI (>15.9 kg for normal weight, >11.3 kg for overweight, and >9.1 kg for obese categories) [16]. Weight by gestational age of the patients was calculated as large for gestational age (LGA), appropriate for gestational age (AGA), and small for gestational age (SGA) [17].

To evaluate the radiation exposure rate, all mothers were questioned about the use of mobile phones, microwaves, computers, and television during pregnancy. For the mobile phone-related radiation, we recorded the average call time per day, time of application use, the proximity of the phone when not used, use of hands-free equipment, and the models of cellular phones. The distance between the mother and mobile phone for most of the day was classified as <50 cm or ≥50 cm [11, 18]. The average specific absorption rate (SAR) value was found on the website reported by the manufacturer (https://www.devicespecifications.com/tr). Effective SAR value was calculated by the formula average SAR × call time per day, which was previously reported [11]. Maternal exposure to electrical devices (television, computer, laptop) was defined as at least two h of use per day was defined. Exposure to a microwave oven was defined as at least one min used per day.

### 2.1. Statistical analysis

Demographics and obstetric data were compared between patients with CAKUT and the control group. Continuous variables were reported as mean ± SD for normal distribution and skewed distribution and was reported as median (interquartile range (IQR). The Mann Whitney-U analysis was performed for skewed distribution and nonparametric variables and the student t-test was used for normally distributed variables. The comparison between groups was made using the chi-square test or Fisher exact test for categorical parameters.

Logistic regression was performed to evaluate the association between CAKUT and the control group by calculating odds ratios (OR) and 95% confidence interval (CI). For multivariate analysis, the possible factors determined with univariate analysis were included in the model to detect independent factors for the result. For the evaluation of model fit, the Hosmer-Lemeshow goodness of fit test was used. A backward stepwise analysis was performed to identify the independent risk factors for CAKUT. The Receiver Operating Characteristic (ROC) curve was used to determine the threshold of the effective SAR. A p-value <0.05 was considered statistically significant and analyses were performed with SPSS, version 22.

## 3. Results

**Figure 1:**
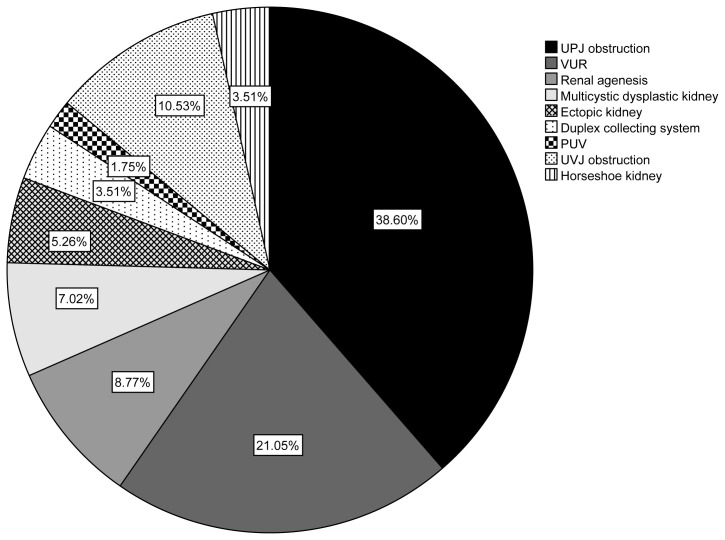
Distribution of CAKUT phenotypes of study participants. UPJ, ureteropelvic junction obstruction; VUR, vesicoureteral reflux; PUV, posterior urethral valve; UVJ ureterovesical junction obstruction

In this research, 114 participants, including 57 cases and 57 controls, were evaluated for analysis. The study population was determined as 57 by using the G power program by taking impact size 0.47 (based on a similar study result [11]) α = 0.05 and power (1-β) =0.80 at a confidence level of 95% [19]. As shown in Figure 1, ureteropelvic junction (UPJ) obstruction 22(38.6%) and vesicoureteral reflux 12(21.0%) were the most common CAKUT phenotype in our case group. There was no significant difference between the ages of parents, gravidity history maternal occupation, and educational level. The rate of having a relative with CAKUT was higher in the case group (11(20%) vs 2(3.5%); p = 0.006). The gender of offspring, birth weight, and gestational week were not statistically different between CAKUT and control groups. Compared to the control, renal and urinary abnormalities were detected more frequently in antenatal USG in the CAKUT group (34(60%) vs. 2(3.5%); p < 0.001). The comparison of the demographics of CAKUT and control groups is demonstrated in Table 1.

Pre-pregnancy chronic disease was more frequent in the CAKUT group (n = 15(26.3%) vs. n = 7(12.3%), p = 0.06). The most common chronic maternal diseases were Type 1 diabetes and hypothyroidism, and there was no statistical difference between the groups. Gestational diabetes was the most common gestational disease and it was nonsignificant among the groups. Maternal BMI value during prepregnancy was slightly higher in the CAKUT group (p = 0.05). Compared to control, excessive weight gain more than expected according to prepregnancy BMI was more common in mothers of the CAKUT group (27(47.4%), 12(21.1%); p = 0.011). Preconception use of the folic acid rate was significantly different between groups (13(22.2%) vs. 24(42.1%); p = 0.028). The rate of using multivitamins was more common in the control group, but there was no significant difference between the groups (36(64.3%) vs. 46(80.7%); p = 0.05). The use of additional medicine was significantly higher in the CAKUT group (25(43.9%) vs. 11(19.3%); p = 0.005). Drinking, and smoking prepregnancy and during pregnancy were not common in our population and our study. The passive smoking rate was similar between both groups. Maternal pregestational and gestational diseases and exposures are summarized in Table 2.

As shown in Table 3, most of the CAKUT and control group mothers used mobile phones during their pregnancy. Mobile phones of the participants except one were all smartphones. Compared to the control, the median call time per day was significantly longer in the CAKUT group (1(1) vs. 1(0.5); p = 0.001) However, using hours of online applications, use of hand-free equipment and the proximity of mobile phones at a close distance (<50 cm) during pregnancy was not different between groups. The median effective SAR value was statistically higher in the case group (1.38(1.33) vs. 0.93(0.86); p = 0.007). The use of other electrical appliances such as television, microwave oven, computer, and wireless internet was not different between the groups.

**Figure 2:**
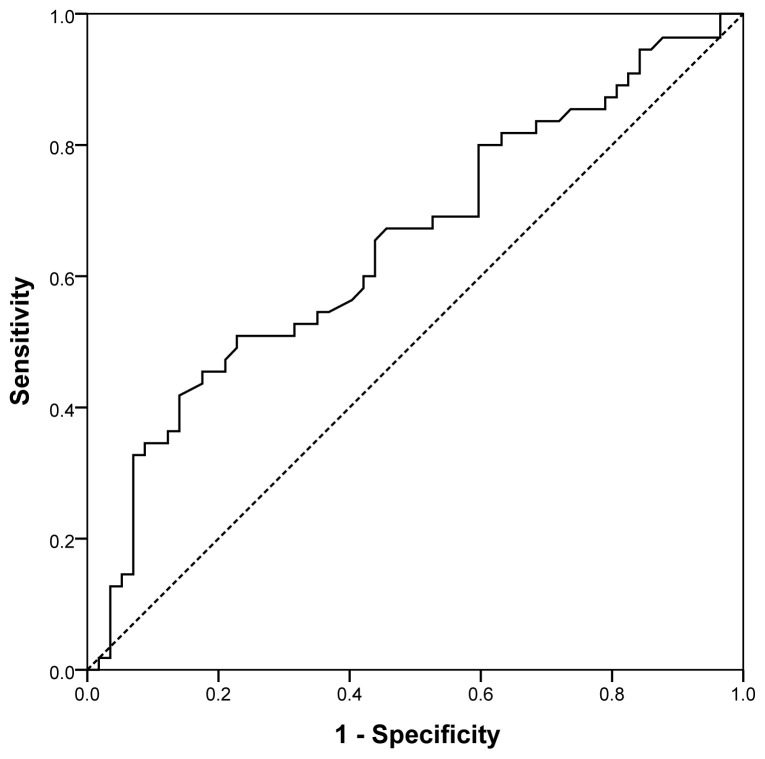
ROC curve of the effective specific absorption rate (SAR) for predicting congenital anomalies of the kidney and urinary tract (CAKUT).

Eight variables in univariate analysis with a p-value <0.1 were included in the stepwise backward logistic regression analysis to find the most predictive multivariable model (CAKUT in relatives, pregestational chronic disease, prepregnancy BMI, weight gain during pregnancy, preconception folic acid supplementation, multivitamin use, taking additional medicine and effective SAR value). As shown in Table 4, multivariate logistic regression analysis revealed a significant association between excess weight gain during pregnancy according to BMI (excessive weight gain OR: 4.4, CI 95%1.4–14.2; p = 0.012) and CAKUT. Folic acid use before pregnancy seemed to be protective in the development of CAKUT (OR: 0.29, CI 95% 0.11–0.79; p = 0.016). A higher effective SAR value as an indicator of EMF exposure to the mobile phone was found to be related to 1.7-fold increased risk of occurrence of CAKUT (OR: 1.7, CI 95%1.1–2.9; p = 0.03). As shown in Figure 2, the ROC curve revealed the cut-off value of effective SAR for predicting CAKUT as 1.13 W/kg (60% sensitivity, 58% specificity, area under curve (AUC): 0.65, 95% CI 0.55–0.75; p = 0.007).

## 4. Discussion

This case-control study evaluated the possible risk factors for the development of CAKUT. Our results revealed excess weight gain according to BMI during pregnancy was related to an increase in the risk of having a baby with CAKUT. Preconception folic acid use was observed to be protective for CAKUT. Unlike previous epidemiological studies, we also questioned the possible effect of mobile phone-related EMF on the development of CAKUT. We observed an association between longer call times during pregnancy and CAKUT. Higher effective SAR values seemed to be an independent risk factor for the development of CAKUT.

In previous studies, many pregestational and gestational risk factors were evaluated for involvement in CAKUT and other possible associated anomalies. Pregestational maternal diabetes mellitus, gestational maternal diabetes mellitus, overweight, and obesity are reported to be associated with CAKUT [5,7]. More specifically, pregestational diabetes mellitus was reported to be associated with renal dysplasia/aplasia. Gestational diabetes and maternal obesity were more often seen in mothers of cases with obstructive uropathies [6, 7]. In this study, we could not show any relationship between a diagnosis of maternal diabetes and CAKUT due to the limited number of patients. Maternal obesity is associated with hyperglycemia and different metabolic abnormalities, so it may increase the frequency of congenital anomalies [20]. In our study, the prepregnancy BMI was higher in the CAKUT group which was consistent with previous studies [6, 21]. Excess weight gain during pregnancy and its association with the risk of CAKUT have been investigated in a limited number of studies. In our study, weight gain during pregnancy in the CAKUT group was higher than expected according to pregestational BMI values (p = 0.011). In a recent study, excess weight gain during pregnancy and increased weight gain/prepregnancy weight ratio were reported to be associated with the occurrence of CAKUT [21].

In our study, periconceptional use of folic acid supplements was preventive against genitourinary birth defects, consistent with previous studies [22–24]. In Turkey, only folate supplementation is recommended for women with a low risk of NTD who are planning pregnancy. However, women of low socioeconomic status are less likely to take folic acid supplements and have low awareness of the importance of supplementation [25]. In contrast, in the USA and Canada, folic acid supplementation was observed to reduce neural tube defects but seemed to be associated with an increasing trend in genitourinary birth defects [26, 27]. Consistently, in a recent study, preconception use of folic acid supplements was reported to slightly increase the risk of CAKUT [6]. They suggested that the reduction in genitourinary birth defects was achieved with a multivitamin supplement containing folic acid rather than folic acid alone [22, 23].

In the current study, the relationship between EMR exposure during pregnancy and the birth of a baby with CAKUT was investigated for the first time in the literature. We observed that the daily call time of the mothers of CAKUT cases was significantly longer than the control. Our results revealed that the location of the mobile phone when not in use did not differ between groups. The effective SAR value, which includes the average call time per day and the SAR value of the mobile device, was higher in the CAKUT group. Mahmoudabadi et al. determined an association between effective SAR value and increased risk of spontaneous abortion [11]. In a recent study, Zhao et al. observed that mothers exposed to electrical appliances during early pregnancy were more likely to give birth to infants with congenital heart disease [9]. In addition, the authors found that prolonged use of mobile phones increases the risk of congenital heart defects [9]. Consistent with our results, an epidemiological study found that maternal proximity to extremely low-frequency EMF was not associated with the risk of birth defects. The prevalence of birth defects was reported to be slightly higher in pregnant women who lived within 200 m of transmission lines and transmission stations, but the risk did not vary between 50 m and 200 m [28]. Many studies are needed to elucidate the impact of EMF exposure.

EMF can generate biological stress and free radicals that can predispose a sensitive population such as a fetus to congenital birth defects and tissue and cellular damage [29]. The power density of EMF radiation decreases up to two orders of magnitude as the distance from the phone increases up to 48 cm [18]. SAR values indicate the power absorbed by particular body tissue, corresponding to 1 g or 10 g of body tissue emitted by a mobile phone and it is measured in watts per kilogram (W/kg). The Federal Communications Commission (FCC) of the United States limits general exposure to cellular phones is a SAR value of 1.6 watts per kilogram (1.6 W/kg) [30]. The mean SAR levels of the CAKUT group were greater than the control, but the difference was not significant (p = 0.18). Effective SAR, a variable that includes call time, was significantly higher in the study group (p = 0.007) and appears to be associated with the risk of having a child with CAKUT (OR: 1.7, CI 95%1.1–2.9; p = 0.03). To our knowledge, this is the first study that evaluates the effects of EMF exposure to mobile phones on CAKUT.

The limitations of our study should be addressed. Our study population includes fewer patients than in previous studies investigating the etiology of CAKUT. We collected preconception and prenatal data based on the pieces of information given by the mothers to the questions in the questionnaires during the face-to-face interviews. The time between birth and completion of the questionnaire was similar and we thought that this was not likely to cause recall bias. We asked about the average phone time of the mothers, but we could not reach their bills for the exact hours of call times. None of our patients were screened for CAKUT-related genes. As for strengths, before evaluating the effect of indirect results of EMF, all potential risk factors were evaluated in detail and included in the stepwise regression analysis. Our study was conducted during the COVID-19 pandemic. We thought that we worked with a homogeneous population, as there were individuals living in the same city, with similar cultures, and experiencing the same restrictions.

## 5. Conclusions

Our study suggested that the lack of folic acid supplementation in the prepregnancy period and excess weight gain during pregnancy play a role in the development of CAKUT. This study highlights that mobile phone-related EMF exposure during pregnancy may be associated with an increased risk of CAKUT in offspring. In the developing world, the risks brought by changing habits and lifestyles are also diversifying, and more attention should be paid to the side effects of EMF exposure during pregnancy. Limiting call times seems to be a good way to reduce EMR exposure, especially during pregnancy. Studies with more participants will provide further insights regarding the effect of EMF on the development of kidneys and the urinary tract.

## Figures and Tables

**Table 1:** Comparison of maternal and paternal demographics by univariate analysis between case and control groups.

Variables	CAKUT (n = 57)	Control (n = 57)	p-value
Maternal characteristics			
Maternal age, year	29.3 ± 6.0	27.9 ± 5.8	0.22
Maternal age group, y(%)			
Maternal age <20	3(5.3%)	5(8.8%)	0.63
Maternal age 20–34	41(71.9%)	42(73.7%)	
Maternal age ≥ 35	13(22.8%)	10(17.5%)	
Maternal education (%)			
Low	10(17.5)	12(21.1)	0.51
Intermediate	32(56.1)	35(61.4)	
High	15(26.3)	10(17.5)	
Maternal occupation (%)			
Housewife	49(86.0)	43(76.8)	0.21
Employee	8(14.0)	13(23.2)	
Consanguineous marriage (%)	10(17.5)	6(10.5)	0.28
CAKUT in relatives (%)	11(20.0)	2(3.5)	**0.006**
Gravidity history (%)			
1	18(31.6)	18(31.6)	1.0
> 1	39(68.4)	39(68.4)	
Vaginal delivery (%)	23(40.4)	26(45.6)	0.57
Paternal characteristics			
Paternal age, year	32.6 ± 6.31	31.9 ± 5.71	0.34
Paternal age group, y (%)			
<20	0	0	0.34
20–34	32(56.1)	37(64.9)	
±35	25(43.9)	20(35.1)	
Offspring characteristics			
Male (%)	35(61.4)	26(45.4)	0.09
Renal abnormality in antenatal ultrasound (%)	34(60)	2(3.5)	**<0.001**
Gestational week <37 (%)	11(19.3)	17(29.8)	0.12
Birth weight (g)	3120 ± 603	2993 ± 637	0.28
Birth weight (%)			
SGA	4(7.0)	4(7.0)	0.55
AGA	43(75.4)	47(82.5)	
LGA	10(17.5)	6(10.5)	
The time between childbirth and filling out the questionnaire, month	6.1 ± 5.5	5.7 ± 6.6	0.73

CAKUT, congenital anomalies of the kidney and urinary tract, SGA, small for gestational age, AGA, appropriate for gestational age, LGA, large for gestational age

p < 0.05

**Table 2:** Comparison of possible risk factors by univariate analysis for CAKUT in pregnancy between case and control groups.

Variables	CAKUT (n = 57)	Control (n = 57)	p-value
Pregestational chronic disease (%)	15(26.3)	7(12.3)	**0.06**
Type 1 diabetes	4(7.0)	2(3.5)	0.68
Hypothyroidism	7(12.3)	2(3.5)	0.16
Others	4(7.0)	3(5.3)	0.7
Gestational disease (%)	8(14.0)	11(19.3)	0.45
Gestational diabetes	6(10.5)	6(10.5)	0.5
Hypertension	2(3.5)	5(8.7)	
Prepregnancy BMI in kg/m^2^	27.1 ± 4.3	25.2 ± 5.68	0.05
Weight gain during pregnancy (%)			
Inadequate	13(22.8)	22(38.6)	**0.011**
Recommended	17(29.8)	23(40.4)	
Excessive	27(47.4)^a^	12(21.1)	
Folic acid use (%)	52(91.2)	51(89.5)	0.75
Preconception use of folic acid	13(22.8)	24(42.1)	**0.028**
Multivitamin use (%)	36(64.3)	46(80.7)	0.05
Taking additional medicine (%)	25(43.9)	11(19.3)	**0.005**
Smoking before pregnancy (%)	5(8.8)	2(3.5)	0.43
Smoking during pregnancy (%)	3(5.3)	1(1.8)	0.61
Passive smoke (%)	27(47.4)	26(46.6)	0.85
Drinking	0	0	

CAKUT, congenital anomalies of the kidney and urinary tract, BMI, body mass index p < 0.05

**Table 3:** Comparison of the use of mobile phones during pregnancy between CAKUT and control groups.

Variables	CAKUT (n = 57)	Control (n = 57)	p-value
Mobile phone use(%)	55(96.5)	57(100)	0.5
Mobile phone time (h)*	3(4)(1–10)	3(2.5)(0.2–8)	0.75
Call time per day (h)*	1(1)(0.25–5)	1(0.5)(0.2–6)	**0.001**
Use of online application (h) *	1.5(3.6)(0–15)	1.5(2.0)(0–7)	0.77
Use of hands-free equipment(%)	12(21.8)	11(19.3)	0.74
Location of phones not in use <50 cm (%)	34(61.8)	37(64.9)	0.73
SAR value of the phone(W/kg)	1.1 ± 0.43	0.98 ± 0.36	0.18
Effective SAR (SAR x call time)*	1.38(1.33)(0.2–4.5)	0.93(0.86)(0.15–5.4)	**0.007**
Wireless in home/work(%)	30(54.5)	35(61.4)	0.46
Watching TV(%)	29(52.7)	36(63.2)	0.52
Use of microwave oven (%)	8(14.5)	17(29.8)	0.07
Use of computer (%)	4(15)	14(26.4)	0.34

CAKUT, congenital anomalies of the kidney and urinary tract, EMF, electromagnetic field, SAR, the specific absorption rate, TV, television

* Median (IQR)(min–max)

p < 0.05

**Table 4:** Multivariate logistic regression analysis* the risk factors associated with CAKUT.

Variables	OR	CI%95	p-value
Preconception use of folic acid	0.29	0.11–0.79	**0.016**
Taking additional medicine	4.9	1.7–13.9	0.06
Weight gain during pregnancy			
Inadequate	Reference	Reference	-
Recommended	1.4	0.43–4.3	0.59
Excessive	4.4	1.4–14.2	**0.012**
Effective SAR value	1.7	1.1–2.9	**0.03**

CAKUT, congenital anomalies of the kidney and urinary tract, SAR, the specific absorption rate

* Eight variables in univariate analysis with a p-value < 0.1 (CAKUT in relatives, pregestational chronic disease, prepregnancy BMI, weight gain during pregnancy, preconception use of folic acid, multivitamin use, taking additional medicine, effective SAR value) were included in the stepwise backward logistic regression model. CAKUT in relatives, pregestational chronic disease, prepregnancy BMI and multivitamin use were removed from the analysis in the early steps.

p < 0.05
